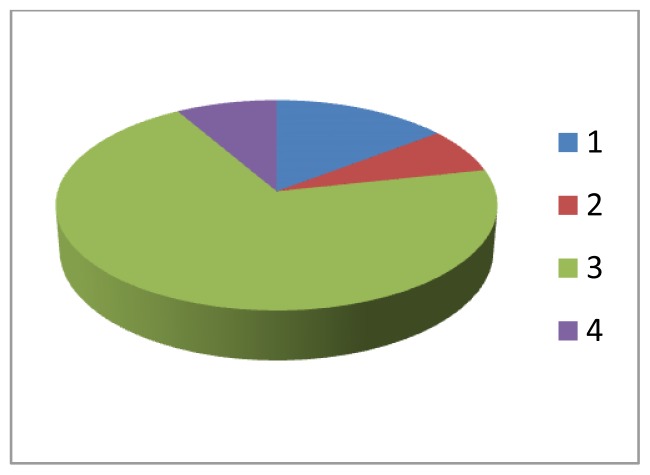# Whence cometh the revolution

**Published:** 2015-04-20

**Authors:** Marcel D’Eon

**Affiliations:** University of Saskatchewan, Saskatoon, Saskatchewan

Seldom does a disruptive technology just appear on the scene like an intergalactic hitchhiker on a meteor or asteroid. The smart phone might seem to be one of those. It revolutionized the way we think about and conduct our lives. It’s really not a phone at all but a mini-computer with which we make the occasional real-time voice call. Problem-based Learning (PBL) and the Multiple Mini Interview (MMI) are two such recent disruptive technologies in medical education. The seemingly abrupt successes and stunning changes related to these two innovations (and the smart phone) may blind us to the long and hard road that led up to them. The MMI grew out of decades of experience with OSCEs and the unavoidable and mounting evidence that the standard interview panel was a waste of time. Similarly with PBL, the adult learning movement (however wrong it may have been) had been gathering steam while multiple papers and commentaries documented and lamented the prevalence of mind-numbing lectures in medical schools classrooms across the world. PBL and the MMI were, as was the smart phone, products of a long line of previous innovations. So it is with a new and disruptive way of representing curriculum being developed at the University of Saskatchewan.

Scholars at the U of S have concluded that a representation of the curriculum which takes into account the full weight of the courses as felt by students and not just the assigned space that these courses occupy in the schedule is more accurate and helpful. What defines the relative place of various components of the curriculum, in many ways the *de facto* importance of those components (bio-medical sciences, clinical skills, clinical decision-making, social and behavioural sciences and humanities among others) is not only the time allocated in the schedule of curricular activities but the weight that those courses exert on the medical students: how much time and energy students devote to each of the curricular areas. That felt or experienced weight, to speak metaphorically, gives an accurate measure of the footprint that the course makes in the curriculum. For example, some of the clinical skills and social science courses are not as dense or difficult as many of the clinical and basic science courses. Students strategically invest more time and energy in those courses that are harder and heavier creating a distortion in the designed curriculum to create the experienced curriculum. Below are two pie graphs that represent this phenomenon.

Graph A represents the designed allocation of relative importance of four components (in this case they are courses) using only one dimension: time in the schedule. Graph B is the multidimentional representation curricular footprint of those same courses, their weight (time and effort expended in attending classes, completing assignments, and studying for tests and exams). Notice that the relative size of the components has changed dramatically. No longer do we have our original designed allocation of importance but something quite different.

Researchers at the University of Saskatchewan are planning to explore which factors or dimensions contribute to the weight of each course and to what extent to develop a more accurate and valid model. But already, having informally validated this innovation, we are wondering how we are to respond. It is shaking some of us up quite a bit, and disrupting our previous conceptions of our curriculum.

Like PBL and the MMI, this disruptive technology was more evolutionary than revolutionary. We knew that some courses were heavier and others lighter and that our students responded strategically, spending more time and energy on some and less on others. We also knew much about cognitive load and its role in learning at the individual level. Then those ideas came together in a useful synthesis that promises productive discomfort as a necessary prelude to major change. At least that is our hope.

The papers in this issue each contribute a building block to the knowledge and intelligence of our medical education community and may eventually lead to a breakthrough, a disruption in the tired and true ways of medical education.

Nguyen, Patenaude, Gagnon, Deligne, and Bouthillier report their findings in ‘Simulation-based assessment of clinical competence for large groups of medical students: a comparison of auscultation sound identification either with or without clinical context in a multiple-choice test.’ They tested two different scenarios for assessing a student’s ability to identify heart and lung sounds: one with the sounds alone and the other accompanied by clinical vignettes. They found statistically significant differences between scores of first, second, fourth year students and residents when clinical vignettes were included. Perhaps a simple multiple-choice test to assess recognition of simulated auscultation sounds incorporated into clinical vignettes is a more valid assessment than just presenting the sounds alone. How far will this take us?

Beagan, Fredericks, and Bryson advocate for further discussion and development around the formation, education, and training in their article ‘Family physician perceptions of working with LGBTQ patients: physician training needs.’ They found that some physicians disagreed that treating everyone as a unique individual optimizes care. Some participants believed that knowing and responding to biological and socio-cultural group membership improved care and some did not. The authors argue for a balanced approach that incorporates both group membership and individual considerations into care for LGBTQ patients. I’m sure this is just the latest in an on-going debate about a tension that may never be entirely resolved. Is there a dialectical solution and if so, whence shall it come?

Margolick, Kanters, and Cameron in ‘Procedural skills training for Canadian medical students participating in international electives’ note that medical students returning from electives abroad often express concern about doing medical procedures that they feel are beyond their level of training. Using surveys to collect data from 26 medical students, they found no evidence that students were performing procedures for the first time, but also discovered a need for additional pre-departure training in several procedural skills. Is there an ethical or educational dilemma demanding some attention here?

Millar, Malcolm, Cheng, Fine, and Wong in ‘Frontline over ivory tower: key competencies in community-based curriculum’ used a modified Delphi technique to determine the competencies required for a community endocrinology curriculum. The experts included endocrinology program directors, community endocrinologists, endocrinology residents and recent endocrinology graduates. They agreed that the community setting was considered to be the best place to learn the “Manager” role but not the best place to learn “Medical Expert.” Community settings certainly have potential to deliver valuable training in residency. When and in what ways will we make more and better use of these valuable learning environments? From some of our American neighbours to the south (Reader, Fornari, Simon, and Townsend) we have ‘Promoting faculty scholarship – an evaluation of a program for busy clinician-educators’. They describe a funded scholarship development program for an urban department of family medicine. Ten participants reported that protected time, coaching by a coordinator, peer mentoring, engagement of project leaders, and involvement of a visiting professor increased their confidence and ability to apply research skills. Academic presentations, publications, and new educational leadership positions followed participation in the program for some of the ten scholars. The situation they document is common and their program promising but who will take the next step and what might that be?

Deonandan and Khan, in ‘Ethics education for pediatric residents,’ conducted a structured literature review to describe ethics education in pediatric residency programs and to suggest possible directions for improvement. They found that current training seems insufficient to meet the real life ethical challenges experienced in actual practice, especially in palliative care and the commission of clinical errors. They recommend an interdisciplinary team approach to ethics training spread over a physician’s entire career. With physician assisted suicide on the horizon, robust ethics training now seems more important than ever, but will we respond?

‘Realism of procedural task trainers in a pediatric emergency medicine procedures course’ by Shefrin, Khazei, and Cheng engaged physicians and trainees in a daylong procedural training course that utilized commercially available and homemade task trainers to teach pericardiocentesis, chest tube insertion, cricothyroidotomy and central line insertion. They found little relationship between cost of the trainers, their perceived realism, and learning utility. They recommend that future courses should carefully consider how the features of task trainers align with the procedural skills being taught balanced against their cost. How will this affect our love affair with expensive and flashy technology?

Where will the studies and ideas found in this issue of CMEJ take us? Well, that’s up to you to create the next disruptive technology and bring us a new breakthrough. That could be as simple as a multidimensional representation of the curricular footprint or as complex as PBL or the MMI. Who knows whence cometh the revolution?

## Figures and Tables

**Graph A:**
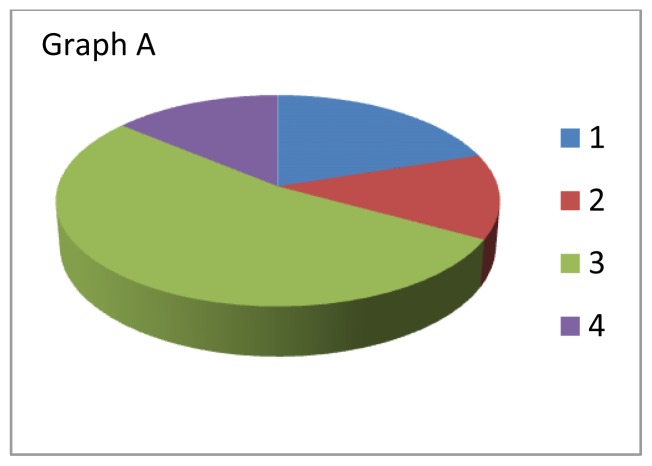


**Graph B:**